# Knowledge, Barriers, and Predictors of Colorectal Cancer Screening in an Appalachian Church Population

**Published:** 2006-09-15

**Authors:** Irene Tessaro, Carol Mangone, Irfan Parkar, Vivek Pawar

**Affiliations:** Community Health Initiatives, School of Nursing, West Virginia University; Community Health Initiatives, School of Nursing, West Virginia University, Morgantown, WVa; Winthrop Hospital, Mineola, NY; Pharmaceutical Systems and Policy, School of Pharmacy, West Virginia University, Morgantown, WVa

## Abstract

**Introduction:**

This study examined knowledge about and barriers to colorectal cancer screening and predictors of screening adherence among members from 16 Appalachian churches as part of a larger study on the prevention and early detection of colorectal cancer.

**Methods:**

Baseline data were collected on 839 respondents aged 50 years and older through a self-administered survey, and 23 focus groups were conducted with 205 church members.

**Results:**

Survey results showed that older age, male sex, being current for other cancer screening, being physically active, having perceived support from others for screening, better provider communication, knowledge about screening guidelines, greater perceived susceptibility to colorectal cancer, and a family history of the disease were predictors of screening adherence. Major barriers to screening in both surveys and focus groups were failure of providers to recommend screening, lack of knowledge about the need for screening, and the belief that screening was not necessary without symptoms. Fear of cancer, lack of knowledge about screening methods other than colonoscopy, reliance on physicians for screening information, and the need for people to feel at risk for screening to occur were other findings from the focus groups. Focus groups supported survey findings and provided further insights.

**Conclusion:**

Several factors predictive of colorectal cancer screening in this study can be modified through educational interventions. Recognizing and changing risk factors for colorectal cancer, raising awareness of screening guidelines, and encouraging adults aged 50 years and older to discuss screening with their health care provider could increase colorectal cancer screening.

## Introduction

Colorectal cancer is the third most commonly diagnosed form of cancer and accounts for approximately 10% of cancer deaths each year. In 2006, an estimated 148,610 new cases of colorectal cancer are expected to occur (1). Early detection is key; the 5-year survival rate is 90% when colorectal cancer is diagnosed at an early localized stage (1). Regular screening with fecal occult blood test (FOBT) or endoscopy (sigmoidoscopy or colonoscopy) can decrease mortality from the disease by 50% or more through early detection and removal of adenomatous polyps or detection at an early stage when the cancer is most curable (2).

The U.S. Preventive Services Task Force guidelines recommend colorectal cancer screening for all individuals aged 50 years and older by annual FOBT and/or by sigmoidoscopy every 5 years, colonoscopy every 10 years, or double-contrast barium enema every 5 years (3). Data from the 2004 Behavioral Risk Factor Surveillance System showed that 57.3% of U.S. adults aged 50 years and older reported having had either an FOBT within the past year or a lower endoscopy in the last 10 years, up from 54.4% in 2002. For FOBT, the percentage is 18.7%, and for lower endoscopy, 50.6%. In Appalachia, West Virginia rates are lower at 19.9% for FOBT, 42.3% for lower endoscopy, and 51.6% for either test (4). Screening rates are increasing but are still below those for other cancer screening tests.

Factors that seem to facilitate screening include higher income, higher education, older age, and male sex (5,6); strong social ties and supportive relationships (7,8); better health care provider communication (9,10); and a physician's recommendation for testing (5,11,12). Preventive health behaviors such as regular exercise, a higher intake of fruits and vegetables, and receiving other cancer screening tests have also been associated with colorectal cancer screening (5,6,13). Having a family history of colorectal cancer is strongly associated with having a colonoscopy (5,6,14).

Clinical and population-based studies of barriers to colorectal cancer screening have reported perceptions of testing as embarrassing (12,14,15), time consuming (15,16), and unnecessary in the absence of symptoms (5,12,14,15). Cultural beliefs about cancer are important in adherence to screening (17,18), particularly fear of finding cancer (12,19). Several qualitative studies have found that overall, people were poorly informed about colorectal cancer (12,20).

This study examines knowledge of and barriers to colorectal cancer screening and predictors of adherence to screening guidelines in an Appalachian church population aged 50 years and older. Data are from baseline surveys and focus groups conducted as part of a larger study evaluating the independent and combined effects of two intervention strategies (parish nurses, natural helpers) on primary and secondary prevention of colorectal cancer in an Appalachian church population.

## Methods

### Church recruitment

Sixteen churches were recruited from the Ohio Valley region of western West Virginia during 2002 to 2003. A list of 708 churches from the seven predominant denominations in the Appalachian region (Baptist, Lutheran, Catholic, Methodist, Presbyterian, Church of Christ, and Nazarene) was obtained from several sources (regional offices of the various denominations, Web sites, telephone books, church contacts). Churches were first contacted by telephone and then, if needed, by mail to determine study eligibility. To be eligible, a church had to have at least 100 active members on its roster and either a parish nurse or health ministry. Of the 708 churches, 143 were not reached after three telephone attempts, a letter, and a 2-week follow-up reminder. Of the 565 churches reached, 376 (67%) did not have 100 active church members, and 115 (20%) did not have a parish nurse or health ministry. Seventy-four churches (13%) met study criteria for random selection; 25 had a parish nurse, and 49 had a health ministry. Churches were separated into two clusters (parish nurse, health ministry) and randomly ordered for recruitment. Churches from each cluster were contacted by telephone in order of randomization to determine interest in learning more about the study. One parish nurse church and 10 health ministry churches could not be reached after repeated attempts and were dropped from further consideration. An initial meeting with 16 randomly selected churches was arranged to present the project to the pastor, priest, or associate pastor and parish nurse or members of the health ministry. Churches varied according to the time it took to commit to the project, ranging from the time of the visit to several months. All churches visited agreed to participate in the study. It took 12 months to finish recruitment; most churches were recruited within 6 months.

### Data collection procedures

An advisory group, consisting of members of existing committees within the church, provided guidance on data collection. Project staff worked with each church advisory group to arrange the best times and church setting for data collection. To encourage church members to participate in the survey, pastors made announcements during church services, research staff gave presentations about the project at church services, announcements were placed in church bulletins, and the advisory group discussed the project individually with church members. All consenting adults (aged 18 and older) were eligible to participate, but special effort was made to recruit active church members (regular church attendees) and those aged 50 and older. Respondents were self-selected and completed a 74-item self-administered survey at the church after services or at other designated times. Survey data were collected on average at 3.5 months (range, 2–7 months) from the church's agreement to participate. This study was approved by the institutional review board at West Virginia University.

### Survey measures and analyses 

The survey assessed sociodemographic characteristics, preventive health care, colorectal cancer screening, and health-promoting behaviors. The sociodemographic characteristics included sex, age, marital status, educational level, employment status, annual household income, and type of coverage for health care costs. Preventive health care assessed a health checkup, mammography in the last 2 years for women, and prostate-specific antigen (PSA) in the last 2 years for men. Communication with a health care provider was assessed with three questions about how often respondents' health care provider gave them enough information to make good decisions about their health and involved them in decisions about their health care and how often they were comfortable asking their doctor for tests or information (always, almost always, sometimes, rarely, or never for each question). These three items were summed to form a scale (Cronbach α = 0.77; mean [± SD] = 9.28 [± 2.45]; range, 0–12).

**Colorectal cancer screening**

Respondents were asked if they had ever had each of the screening tests for colorectal cancer (yes or no) and whether they had the test(s) in accordance with the U.S. Preventive Services Task Force guidelines (yes or no): FOBT (1 year), sigmoidoscopy (5 years), colonoscopy (10 years), or double contrast barium enema (5 years). A brief description of the test preceded each question. Respondents were also asked how often (in years) they had heard that they should get each test. Those who reported never having had a colorectal cancer screening test were asked what had prevented them from getting tested (never heard of the tests, did not know needed one, doctor never recommended, too busy, financial reasons, no need because no problems, afraid of finding a problem, not old enough, test uncomfortable, test embarrassing). Family history of colorectal cancer was assessed with the question, "Has your mother, father, or any of your brothers or sisters ever had colorectal cancer?" (yes or no). Perception of risk for colorectal cancer was assessed with the question, "Compared to others your age, what do you think your chances are of getting colorectal cancer?" (more than average, about average, or less than average). Respondents were also asked how much they could count on those close to them for support and help if they wanted to be screened for colorectal cancer (a lot, somewhat, very little, or not at all).

**Health-promoting behaviors**

Level of physical activity was calculated based on responses to a modified Community Healthy Activities Model Program for Seniors (CHAMPS) physical activity survey designed for older adults (21). This frequency-based checklist included 30 activities with metabolic equivalent (MET) values ranging from 2 to 6 and included recreational and leisure-time activity, occupation-related activity, and home-related activity. Minor modifications included omitting a few of the sedentary activities and adding items related to occupational activity. Participants were asked whether they did each of these activities in a typical week during the last month; those who responded yes were asked how many times and hours each week each activity was done. Participants met physical activity guidelines if they accumulated either 150 minutes of moderate-intensity physical activity (MET ≥3) for 5 or more days per week or 60 minutes of vigorous-intensity physical activity (MET ≥4) for 3 or more days per week. Intake of at least five fruits and vegetables per day was calculated using a 34-item food frequency checklist adapted from an instrument originally validated from National Health and Nutrition Examination Survey (NHANES) II data (22) and adapted in other studies (23,24). Responses for each of 11 fruit and vegetable items included 3 or more per day, 2 per day, every day, 2 to 4 per week, once a week, 1 to 3 times per month, and never or almost never. Weight and height were self-reported. Body mass index (BMI) was calculated as weight (lb)/height (in)^2^ multiplied by 703. A BMI less than 25.0 was categorized as normal weight, a BMI from 25.0 to 29.9 was categorized as overweight, a BMI from 30.0 to 34.9 was categorized as obese, and a BMI of 35 or greater was categorized as morbidly obese.

Frequencies and Χ^2^ analyses were conducted for descriptive statistics and the bivariate analysis of factors predictive of colorectal cancer screening. Missing data were excluded for all analyses, and sample size varied for the different tests. All analyses were performed using SAS version 9.1 (SAS Institute Inc, Cary, NC).

### Focus groups

After baseline surveys were completed, 23 focus groups were conducted in 12 intervention churches to better understand church members' knowledge and perceptions about colorectal cancer prevention and early detection and barriers to and facilitators of behavior change. Two focus groups were conducted in each church, one with men and one with women; one church had a mixed-sex focus group session. Project team members moderated all focus groups. The discussions were conducted in the church and lasted about 1 hour. Groups were tape-recorded, and notes were taken. Observer notes were incorporated into the transcripts. A brief 8-question survey was completed by each participant to profile the group. An open-ended interview script informed by social cognitive theory (25), the health belief model (26), and social support theory (27) guided the discussions. Questions about colorectal cancer focused on 1) knowledge and attitudes about colorectal cancer; 2) knowledge about screening tests; 3) risk factors for colorectal cancer; and 4) barriers to and facilitators of colorectal cancer screening.

Data were transcribed and analyzed using qualitative methods (28). To avoid potential bias and provide objectivity, analysis was conducted separately by two study investigators. Text analysis computer software, Ethnograph version 5.0 (Qualis Research Associates, Colorado Springs, Colo), was used to systematically organize, code, and sort data into categories for interpretation. Data were analyzed first by reading the transcripts and listening to the tapes, then noting patterns. Comparisons were made between the men's and women's groups. The research team compared similarities and differences in data interpretation and agreed on a final analysis of significant themes.

## Results

### Study sample

A total of 1238 church members completed the self-administered survey, 839 (68%) of whom were aged 50 or older. Fifteen respondents who reported a diagnosis of colorectal cancer were excluded, leaving 824 respondents aged 50 or older for this analysis. Nearly all respondents were white (98%), reflecting the population of West Virginia (29). Two thirds (65%) were women, and more than half (56%) were aged 65 or older. Three fourths (76%) were married. One third (38%) were employed, and more than half (57%) were retired. Virtually all respondents had one or more types of health insurance, most from an employer (70%) or Medicare (52%). Almost everyone had a regular health care provider (98%) and a health visit in the last 2 years (94%). The majority were active members of their church and attended services at least weekly (91%).

Focus group sessions were conducted with 205 church members, 127 women (62%) and 78 men (38%). The majority (87%) were aged 50 and older. All participants were white. Most attended church at least weekly (87%). Almost all respondents reported having health insurance (96%). Fewer than half of those aged 50 and older (45%) had ever had colorectal cancer screening.

### Colorectal cancer screening

An FOBT in the preceding year was reported by 26% of survey respondents; 58% had ever had the test. Twenty-five percent had had a sigmoidoscopy in the previous 5 years; 40% had ever had the test. Forty-two percent reported a colonoscopy in the last 10 years; 49% had ever had the test. Only 10% had had a double-contrast barium enema in the last 5 years. More than half of respondents (61%) had at least one of the screening tests within the recommended time frame; 44% of these had had more than one of the tests, and 52% had had an endoscopy.

Nearly everyone in the focus groups was aware of colonoscopy as a test, and this generated most of the discussion. Participants thought it was the best test for early detection. Little discussion revolved around the FOBT, which was considered by many to be an outdated test, as indicated by these comments:

I had that test 20 years ago. I didn't even know if they do those tests anymore with all the new stuff that's come out. [Men's group]

They used to give you the test kits. They don't do that anymore. . . . That seems to be a thing of the past. [Women's group]

The FOBT was not considered as accurate or as thorough as the colonoscopy. There was also little discussion about the sigmoidoscopy, which was considered more painful and less thorough than the colonosocopy.

### Knowledge of screening recommendations

Most survey respondents reported that they did not know that an FOBT was recommended every year (61%) or that sigmoidoscopy was recommended every 5 years (63%). Almost half of respondents (47%) did not know the recommendations for colonoscopy. For FOBT, 23% knew that the recommendation was every year, and 20% knew that a sigmoidoscopy was recommended every 5 years. Few (4%) knew that a colonoscopy was recommended every 10 years; most (23%) thought the recommendation was every 5 years.

Few focus group participants knew about colorectal cancer screening recommendations, but what was known was discussed in relation to colonoscopy. Generally, participants felt that doctors had different recommendations on the frequency of screening with colonoscopy and that recommendations depended  on symptoms, findings during testing, family history, and other factors such as age. For participants who knew anything about screening recommendations, most thought that a colonoscopy should be done every 3 to 5 years and reported that they received this information mostly from their physicians.

### Barriers to screening

Among participants who had never had colorectal cancer screening, the most frequently cited reasons for not being screened were 1) their physician never recommended the test (68%); 2) they did not know they needed the test (43%); and 3) they saw no need because they were not having any problems (39%). About one fourth had never heard of the FOBT (24%), although fewer had never heard of sigmoidoscopy (17%) or colonoscopy (7%). Less than 10% of respondents mentioned that the test was uncomfortable or embarrassing, they had no time, had financial constraints, or were afraid of finding a problem as reasons for not being screened. There were no differences by age or sex for any of the barriers. However, those who reported that their physician had never recommended the test were more likely to report poorer communication with their health care provider (P = .01).

Fear of cancer — not only colorectal cancer but cancer in general — was a major theme in all focus groups. The view that colorectal cancer was a fatal disease was repeatedly brought up as an issue:

Just the word *cancer* frightens people. We just all assume you die of it. [Men's group]

I think everybody is scared of cancer. And consequently, if you're 70 years old, I'm not sure whether you want to know it or not. Now in your thirties or forties, yeah. [Women's group]

All groups discussed the failure of physicians to recommend screening for colorectal cancer as a major barrier to screening. Unless the patient mentioned screening, it was often not brought up during a health care visit. When there was a recommendation, it was for colonoscopy. Women especially felt that their doctors failed to recommend screening tests. In general, women felt doctors were more attentive to men's health than women's health, particularly older women's health:

I'm near 60, and my doctor has never suggested even the first test, and he always prides himself on being a doctor that is very interested in prevention. [Women's group]

Doctors don't suggest it. When I was younger, my doctor was very careful and thorough, a complete physical every year, complete blood work, and now any complaint I have, "It's just your age." [Women's group]

The cost of screening and lack of health insurance were discussed as major barriers to screening, mainly colonoscopy, in all groups. Participants felt that the cost of screening was unaffordable for those without "good" health insurance:

I mean, there's less and less health insurance and, like, in our region here, the steel mills are cutting back, and people are losing the hospitalization, the retirees and all, and they are going to have to pick this up, and it's expensive, and some are looking and saying do this or not type thing. The last time I checked, a $1000 deductible for my family is $1500 a month. [Men's group]

Lack of knowledge about colorectal cancer or the screening tests was seen as a barrier either because people did not know they needed a test, never had heard of the test, or felt that there was no need because they were not having any problems. Men in particular said they did not want to go to doctors because of the fear of finding something wrong:

Most people you know don't want to find it. So if they don't go and get it checked, they don't have to worry about it. But we all know we should get it checked, but it's the fear factor. [Men's group]

Participants felt that people needed to feel at risk for colorectal cancer to undergo screening. Many did not see themselves at risk if they did not have symptoms or a family history of the disease. Family history and diet were the most recognized risk factors for colorectal cancer, particularly family history. There was little specific discussion about other risk factors such as obesity or sedentary lifestyles.

Several ways to promote colorectal cancer screening were discussed in the focus groups. These included 1) promoting health messages about colorectal cancer through community education; 2) having support from friends, relatives, and others; 3) learning how to be proactive and advocate for one's health by asking about screening; 4) reducing the fear of colorectal cancer through testimonials and encouragement from reliable sources of information such as friends and "common people" who had survived the disease or had screening tests; and 5) receiving better explanations of the screening procedures from a physician.

### Predictors of screening

Relationships between sociodemographic characteristics, preventive health care, health-promoting behaviors, and being current for at least one of the colorectal cancer screening tests are shown in the Table. Survey findings show that individuals significantly more likely to be current with screening guidelines were between ages 65 and 74 years (*P* = .05); were male (*P* = .03); were retired from employment (*P* =.03); had health care costs covered by Medicare (*P* = .003); had had a recent mammogram (*P* < .001) or recent PSA (*P *<.001); had a family history of the disease (*P <* .001); met physical activity guidelines (*P *= .001); perceived themselves to be at more than average risk for the disease (*P* < .001); perceived they had support for screening (*P* = .03); and had knowledge of screening guidelines *(P* < .001). In addition, participants who reported better communication with their health care provider were more likely to be adherent for screening (*t* = 3.77, *P *<.001) (data not shown).

## Discussion

Combining both quantitative (survey) and qualitative (focus groups) research methods can provide a more comprehensive view of a health issue (30) — in this case, the prevention and early detection of colorectal cancer. Some of the survey results have been reported previously (6). Focus group findings helped us gain more insight into, as well as support for, survey results.

Age has consistently been found to predict screening, with rates higher among individuals aged 65 to 74 years than among those aged 50 to 64 years, and with a peak at 75 years (5,6). These survey results were similar. In this study, as well as others, men were more likely to get screening (5). Some explanation for this comes from the women's focus groups, in which the participants discussed their feelings about doctors being less attentive to older women's health.

Preventive health behaviors, such as regular exercise, a higher intake of fruits and vegetables, and having other cancer screening tests, have also been associated with colorectal cancer screening (5,6,13). This study found that women who had had a recent mammogram, men who had a recent PSA, and participants who were more physically active were more likely to be screened for colorectal cancer with colorectal cancer screening as part of a more preventive health orientation.

A family history of colorectal cancer, which has been strongly associated with having a colonoscopy (5,6,14), was a predictor for screening in this study. Individuals with a family history of a particular disease often consider themselves at higher risk for that disease (31). In these as well as other focus groups (21), family history was the most often mentioned risk factor for colorectal cancer. Low perceived susceptibility to colorectal cancer was also found to be a barrier in both surveys and focus groups (32). Lack of awareness, failure of physicians to recommend screening, and the belief that screening is unnecessary without symptoms are the most commonly reported barriers to colorectal cancer screening (11,12). This study found similar barriers in surveys and focus groups.

Health care provider communication has been shown to be an important predictor of screening. Patients in rural areas who reported that they had adequate time to discuss screening with their physicians were found to be more up-to-date with screening (9). Also, in a study of colorectal cancer screening in an African American church population, those who rated their communication with providers as "good" were more likely to be screened for colorectal cancer (10). In this study, respondents who reported better communication with their health care provider were also more likely to report being screened, and those who reported lack of a physician recommendation for screening had poorer communication with their physicians.

The surveys and focus groups both showed that people knew little about screening recommendations. Participants in the focus groups said they relied on their physicians for advice about which test to have and when to have them. They considered colonoscopy the most accurate of the screening tests and this was the test they said was most often recommended by their physicians. For many, the FOBT was a test of the past. A recent study showed that even when FOBT was preferred by patients, physicians referred for endoscopy (33).

Findings from the surveys and focus groups were similar. However, there were inconsistent findings. Although the cost of screening and lack of health insurance were consistently mentioned as barriers in the focus groups, few respondents mentioned them in the survey. In these focus groups, the cost of health care and insurance were not discussed in relation to colorectal cancer screening only but in relation to the broader problems of these issues in the health care system. Also, fear of cancer was a major theme in these and other focus groups (12), but "afraid of finding a problem" was rarely mentioned as a barrier to screening in the surveys.  

There were several limitations to this study. This surveyed church population did not represent all churches in West Virginia, since only those churches that had at least 100 active church members and that had health as part of their mission were recruited for the study. Church members who participated in the study were active members of their church and thus do not represent all members of the church. They may have been more compliant for colorectal cancer screening. Another limitation was that colorectal cancer screening data were self-reported and not verified through health records. Most respondents had some form of health care insurance to cover health care costs, but coverage for colorectal cancer screening was not assessed, so it is unclear what role insurance coverage may have played in screening for colorectal cancer.

Despite these limitations, the study found several factors predictive of colorectal cancer screening in this Appalachian church population that are modifiable through educational interventions. Raising awareness of screening guidelines, recognizing and changing modifiable risk factors, discussing screening with other network members, and encouraging individuals aged 50 and older to initiate a discussion about colorectal cancer screening with their health care provider could increase screening rates.

## Figures and Tables

**Table:** Bivariate Associations with Colorectal Cancer Screening in an Appalachian Church Population (N = 821), 2002–2003^a^ ^b^

** Characteristic**	**No.**	**% With Any Screening^c^(n = 504)**	**Χ^2^ *_df_* (*P* Value)**
**Age, y**			
50-64	364	51.1	Χ^2^_2_ = 6.19 (.05)
65-74	291	66.7	
≥75	166	61.5	
**Sex**			
Female	535	58.7	Χ^2^_1_ = 4.99 (.03)
Male	285	66.7	
**Education**			
Less than high school	44	70.5	Χ^2^_3_ = 1.59 (.66)
High school diploma or GED	269	61.0	
Postsecondary or some college	229	60.7	
College degree or post college	272	61.4	
**Annual household income, $**			
<20,000	117	54.7	Χ^2^_4_ = 2.49 (.65)
20,000-29,999	143	59.4	
30,000-49,999	184	58.2	
50,000-74,999	130	60.0	
≥75,000	100	65.0	
**Employment**			
Full or part time	305	57.1	Χ^2^_2_ = 7.23 (.03)
Retired	459	65.4	
Not employed or have disability	48	52.1	
**Health insurance**			
Employer	575	62.4	Χ^2^_1_ = 0.89 (.35)
Medicare	426	66.2	Χ^2^_1_ = 8.64 (.003)
Self-pay	150	64.0	Χ^2^_1_ = 0.53 (.47)
Other	89	60.7	Χ^2^_1_ = 0.02 (.88)
**Mammogram in last 2 years**			
Yes	473	61.7	Χ^2^_1_ = 16.76 (<.001)
No	59	33.9	
**Prostate-specific antigen (PSA) in last 2 years**			
Yes	232	72.4	Χ^2^_1_ = 18.54 (<.001)
No	53	41.5	
**Family history of colorectal cancer**			
Yes	106	77.4	Χ^2^_1_ = 13.13 (<.001)
No	707	59.0	
**Perception of risk for colorectal cancer**			
More than average	47	87.2	Χ^2^_2_ = 15.95 (<.001)
Average	381	62.7	
Less than average	374	57.5	
**Support for screening**			
A lot	638	62.9	Χ^2^_1_ = 4.82 (.03)
Somewhat/very little	140	52.9	
**Knowledge of screening guidelines**			
Know at least one	299	77.9	Χ^2^_1_ = 54.26 (<.001)
Know none	522	51.9	
**Body mass index, kg/m^2^**			
<25.0 (Normal)	277	62.1	Χ^2^_3_ = 1.55 (.67)
25.0-29.9 (Overweight)	308	61.4	
30.0-34.9 (Obese)	144	63.9	
≥35 (Morbidly obese)	62	54.8	
**Eat at least five fruits or vegetables per day**			
Yes	210	62.4	Χ^2^_1_ = 0.18 (.67)
No	611	61.1	
**At least 150 min moderate or 60 min vigorous activity per week**			
Yes	315	68.3	Χ^2^_1_ = 10.16 (.001)
No	506	57.1	

a Three respondents did not answer the colorectal cancer screening questions.

b Some categories have missing data. Missing data were excluded for all analyses.

c Any screening was defined as a fecal occult blood test (FOBT) in the past year, a sigmoidoscopy within the last 5 years, a colonoscopy within the last 10 years, or a double-contrast barium enema within the last 5 years.

## References

[1] American Cancer Society. Facts and figures.

[2] Walsh JM, Terdiman JP (2003). Colorectal cancer screening: scientific review. JAMA.

[3] (2002). U.S. Preventive Task Force. Summaries for patients. Screening for colorectal cancer: recommendations from the United States Preventive Services Task Force. Ann Intern Med.

[4] (2006). Centers for Disease Control and Prevention (CDC). Increased use of colorectal cancer tests — United States, 2002 and 2004. MMWR Morb Mortal Wkly Rep.

[5] Seeff LC, Nadel MR, Klabunde CN, Thompson T, Shapiro JA, Vernon SW (2004). Patterns and predictors of colorectal cancer test use in the adult U.S. population. Cancer.

[6] Subramanian S, Klosterman M, Amonkar MM, Hunt TL (2004). Adherence with colorectal cancer screening guidelines: a review. Prev Med.

[7] Mitchell-Beren ME, Dodds ME, Choi KL, Waskerwitz TR (1989). A colorectal cancer prevention, screening, and evaluation program in community black churches. CA Cancer J Clin.

[8] Kang SH, Bloom JR (1993). Social support and cancer screening among older black Americans. J Natl Cancer Inst.

[9] Greiner KA, Engelman KK, Hall MA, Ellerbeck EF (2004). Barriers to colorectal cancer screening in rural primary care. Prev Med.

[10] Katz ML, James AS, Pignone MP, Hudson MA, Jackson E, Oates V (2004). Colorectal cancer screening among African American church members: a qualitative and quantitative study of patient-provider communication. BMC Public Health.

[11] Coughlin SS, Thompson T (2005). Physician recommendation for colorectal cancer screening by race, ethnicity, and health insurance status among men and women in the United States, 2000. Health Promot Pract.

[12] Beeker C, Kraft JM, Southwell BG, Jorgensen CM (2000). Colorectal cancer screening in older men and women: qualitative research findings and implications for intervention. J Community Health.

[13] Shapiro JA, Seeff LC, Nadel MR (2001). Colorectal cancer-screening tests and associated health behaviors. Am J Prev Med.

[14] Janz NK, Wren PA, Schottenfeld D, Guire KE (2003). Colorectal cancer screening attitudes and behavior: a population-based study. Prev Med.

[15] Brenes GA, Paskett ED (2000). Predictors of stage of adoption for colorectal cancer screening. Prev Med.

[16] Wardle J, Sutton S, Williamson S, Taylor T, McCaffery K, Cuzick J (2000). Psychosocial influences on older adults' interest in participating in bowel cancer screening. Prev Med.

[17] Scroggins TG, Bartley TK (1999). Enhancing cancer control: assessing cancer knowledge, attitudes, and beliefs in disadvantaged communities. J La State Med Soc.

[18] Paskett ED, Rushing J, D'Agostino R, Tatum C, Velez R (1997). Cancer screening behaviors of low-income women: the impact of race. Womens Health.

[19] Greiner KA, Born W, Nollen N, Ahluwalia JS (2005). Knowledge and perceptions of colorectal cancer screening among urban African Americans. J Gen Intern Med.

[20] Goel V, Gray R, Chart P, Fitch M, Saibil F, Zdanowicz Y (2004). Perspectives on colorectal cancer screening: a focus group study. Health Expect.

[21] Stewart AL, Mills KM, King AC, Haskell WL, Gillis D, Ritter PL (2001). CHAMPS physical activity questionnaire for older adults: outcomes for interventions. Med Sci Sports Exerc.

[22] Block G, Hartman AM, Dresser CM, Carroll MD, Gannon J, Gardner L (1986). A data-based approach to diet questionnaire design and testing. Am J Epidemiol.

[23] Campbell MK, DeVellis BM, Strecher VJ, Ammerman AS, DeVellis RF, Sandler RS (1994). Improving dietary behavior: the effectiveness of tailored messages in primary care settings. Am J Public Health.

[24] Campbell MK, Tessaro I, DeVellis B, Benedict S, Kelsey K, Belton L (2002). Effects of a tailored health promotion program for female blue-collar workers: Health Works for Women. Prev Med.

[25] Bandura A (1996). Social foundations of thought and action: a social cognitive theory.

[26] Strecher VJ, Rosenstock IM, Glanz K, Rimer BK, Lewis FM (1997). The health belief model. Health behavior and health education: theory, research, and practice.

[27] Berkman LF (1995). The role of social relations in health promotion. Psychosom Med.

[28] Patton MQ (1990). Qualitative evaluation methods.

[29] Henry J. Kaiser Family Foundation. West Virginia: population distribution by race/ethnicity, states (2003-2004), U.S. (2004).

[30] Steckler A, McLeroy KR, Goodman RM, Bird ST, McCormick L (1992). Toward integrating qualitative and quantitative methods: an introduction. Health Educ Q.

[31] Tessaro I, Smith SL, Rye S (2005). Knowledge and perceptions of diabetes in an Appalachian population. Prev Chronic Dis.

[32] McCaffery K, Borril J, Williamson S, Taylor T, Sutton S, Atkin W (2001). Declining the offer of flexible sigmoidoscopy screening for bowel cancer: a qualitative investigation of the decision-making process. Soc Sci Med.

[33] Wolf RL, Basch CE, Brouse CH, Shmukler C, Shea S (2006). Patient preferences and adherence to colorectal cancer screening in an urban population. Am J Public Health.

